# Ultrathin carpet cloak enabled by infinitely anisotropic medium

**DOI:** 10.1038/s41598-023-44984-w

**Published:** 2023-10-17

**Authors:** Mohammad Hosein Fakheri, Ali Abdolali

**Affiliations:** Applied Electromagnetic Laboratory, School of Electrical Engineering, Iran University of Science and Technology, Tehran, 1684613114 Iran

**Keywords:** Applied physics, Transformation optics

## Abstract

Thanks to the pioneering studies conducted on the fields of transformation optics (TO) and metasurfaces, many unprecedented devices such as invisibility cloaks have been recently realized. However, each of these methods has some drawbacks limiting the applicability of the designed devices for real-life scenarios. For instance, TO studies lead to bulky coating layer with the thickness that is comparable to, or even larger than the dimension of the concealed object. In this paper, based on the coordinate transformation, an ultrathin carpet cloak is proposed to hide objects with arbitrary shape and size using a thin anisotropic material, called as infinitely anisotropic medium (IAM). It is shown that unlike the previous metasurface-based carpet cloaks, the proposed IAM hides objects from all viewing incident angles while it is extremely thin compared with the object dimensions. This material also circumvents the conventional transformation optics’ complexities and could be easily implemented in practical scenarios. To demonstrate the capability of the proposed carpet cloak, several full-wave simulations are carried out. Finally, as a proof of concept, the IAM is implemented based on the effective medium theory which exhibits good agreement with the results obtained from the theoretical investigations. The introduced material not only constitutes a significant step towards the invisibility cloak but also can greatly promote the practical application of the other TO-based devices.

## Introduction

An invisibility cloak that can conceal objects from the electromagnetic (EM) wave has attracted considerable attentions for centuries, but its realization remained unattainable until the advent of metamaterials. To design such an unprecedented device, various techniques such as, scattering cancelation^1^ and transformation optics^2^, have been proposed. However, the method of scattering cancelation is limited to small hidden objects in comparison to the operative wavelength^1^. From the viewpoint of TO and based on the form invariance of Maxwell’ equations, an initial coordinate system (*i.e.,* virtual domain) is transformed to its counterpart in another arbitrary coordinate system (*i.e.,* physical domain), which results in a direct link between the material properties and metric tensor of the transformed space having the desired EM functionalities^3–6^. However, the materials obtained through this approach are inhomogeneous and anisotropic, which are difficult to be implemented. To obviate the anisotropy issue, the method of quasi-conformal mapping is utilized^7^. Nevertheless, even by performing such a simplifying assumption, this cloak causes a lateral shift of the scattered wave, whose value is comparable to the height of the cloaked object, making the object detectable^8^. Moreover, the mentioned techniques generally yield a bulky coating later with a thickness comparable to, or even larger than the dimension of the concealed object. As the 2D version of metamaterials, metasurfaces can control the EM waves via imparting abrupt phase changes determined by subwavelength elements at the interface and have overcame the certain drawbacks of bulky metamaterials^9–11^. These ultrathin structures have paved the way towards achieving carpet cloaks hiding objects on the ground plane^12,13^. Despite claims about wide-angle metasurface carpet cloak^14^, their functionality is restricted to a specific angular range. Indeed, the cloaking performance is drastically deteriorated out of these incident wave angles. Moreover, the studies by Xu et al.^15,16^ proposes a deterministic approach to achieve full-polarization cloak, a promising innovation for real-world stealth applications. These works present the development of a metasurface skin cloak that operates under any arbitrary polarization state for plane wave incidents. However, it cannot respond effectively to all types of incident waves. This limitation restricts the cloak’s universal applicability and poses a challenge in environments with diverse wave characteristics. Furthermore, the shape of the cloaks is fixed (triangular or trapezoid platform), which may not be suitable for all types of objects. Consequently, for the above-mentioned reasons, one must ask the question of whether there is any alternative way to conceal an object with an ultrathin carpet cloak, regardless of the incident wave angle.

Here, we successfully design an ultrathin all-angle coating layer to conceal objects with arbitrary size and shape located on the ground, based on a special type of material named infinitely anisotropic medium (IAM). It will be demonstrated that the introduced IAM is independent of the concealed object geometry in such a way that if the object shape is changed, one can still use the same IAM. Several numerical simulations are carried out to verify the validity of the propounded invisibility cloak. It is observed that numerical results are in good agreement with the theoretical investigations indicating the validity of the presented approach. As a proof of concept, an ultrathin carpet cloak is realized through the effective medium theory (EMT) by using epsilon near zero (ENZ) metamaterial and NiCo ferrite layer which play the role of required IAM.

## Results

### Theory

**Figure 1:**
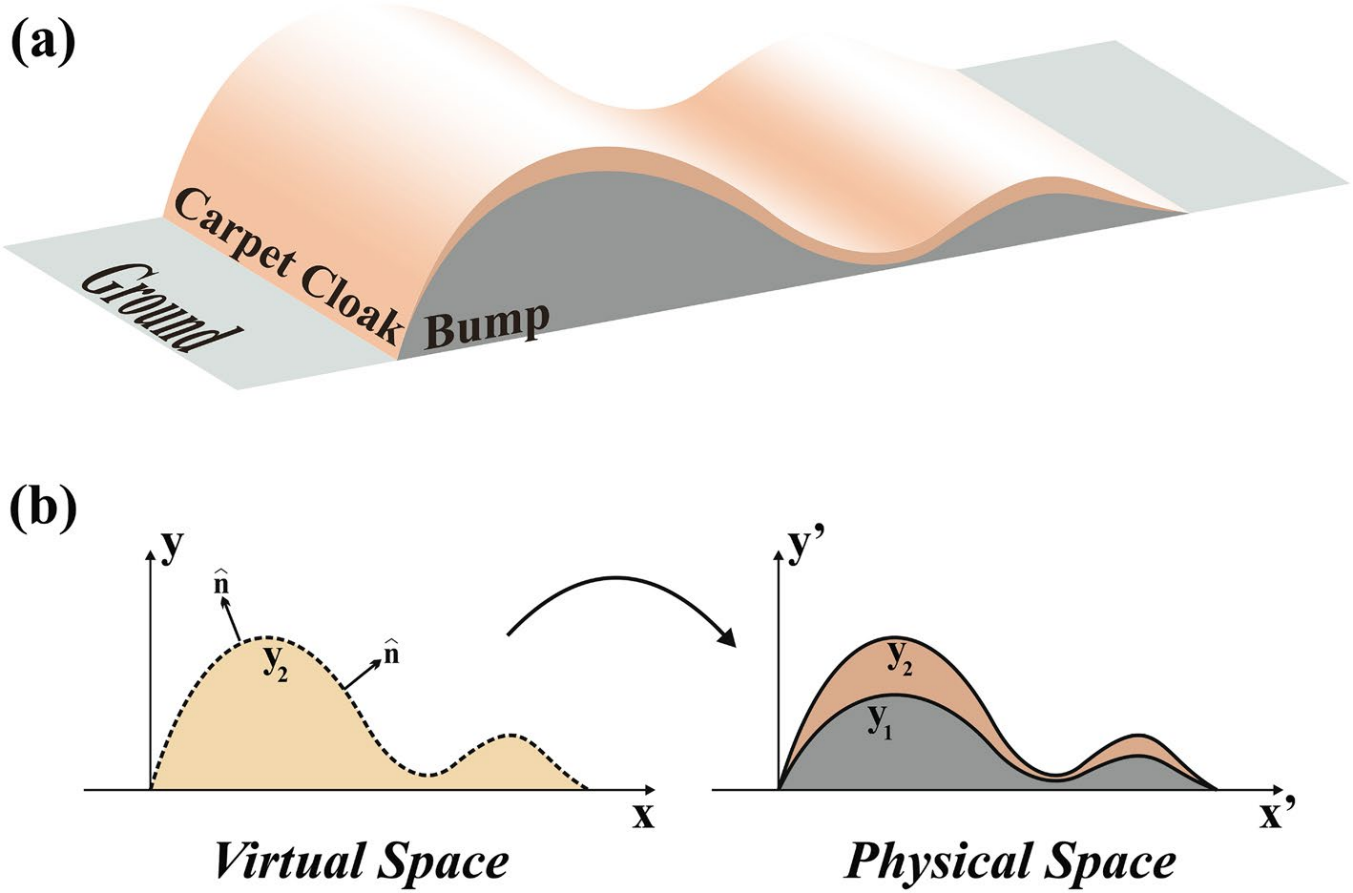
(**a**) Schematic view of the proposed ultrathin carpet cloak for arbitrary shape object. (**b**) Designing a carpet cloak based on coordinate transformation. a cream region in the virtual space is mapped to a brown region in the physical space. ($\hat{n}$ is the optical axis placed perpendicular to the surface $y_1=f(x)$ when $\Gamma \rightarrow 1$).

Figure 1a shows the schematic view of the proposed carpet cloak that conceals the arbitrary shape object with an ultrathin coating layer. Let us start with a straightforward coordinate transformation in the general form. In order to conceal an arbitrary shape object with the curve of $y_1(x)$, the schematic of Fig. 1b is used as the space transformation. As can be seen, a cream region enclosed between the ground $\left( y=0 \right)$ and curve $y_2(x)$ in the virtual space is compressed into the brown region encircled by $y_1(x)$ and $y_2(x)$ in the physical space. Indeed, the ground is transformed to the concealed object boundary with the curve $y_1=f(x)$ while the boundary $y_2=\Gamma f(x)$ is mapped to itself keeping the exterior EM fields unchanged.

The transformation function of such a mapping could be written as $x'=x$, $y'=\alpha y+f(x)$ and $z'=z$ where $\alpha =\left( \Gamma -1 \right) /\Gamma$. According to the coordinate transformation theory, when a space (*x*, *y*, *z*) is transformed into another space $(x',y',z')$ of different shape and size, the permittivity $\overline{\overline{{{\varepsilon }'}}}$ and permeability $\overline{\overline{{{\mu }'}}}$ values in the transformed space are given by $\overline{\overline{{{\varepsilon }'}}}={\Lambda \varepsilon {{\Lambda }^{T}}}/{\det \Lambda }\;$ and $\overline{\overline{{{\mu }'}}}={\Lambda \mu {{\Lambda }^{T}}}/{\det \Lambda }\;$, where $\varepsilon$ and $\mu$ refer to the permittivity and permeability in the original space and $\Lambda ={\partial \left( {x}',{y}',{z}' \right) }/{\partial \left( x,y,z \right) }\;$ indicates the Jacobian transformation tensor^2^. Consequently, the constitutive parameters of the proposed carpet cloak could be expressed as

1 $$\begin{aligned} \frac{\overline{\overline{\varepsilon '}}}{\varepsilon }=\frac{\overline{\overline{\mu '}}}{\mu }=\frac{1}{\alpha } \begin{bmatrix} \ 1 &{} f'(x)&{} 0 \\ f'(x) &{} \alpha ^2+(f'(x))^2 &{} 0\\ 0 &{} 0 &{} 1 \end{bmatrix} \end{aligned}$$

The obtained material of Eq. (1) is inhomogeneous and anisotropic with off-diagonal components of $f'(x)$, which may cause serious difficulties in its realization process. However, as we aim to design an ultrathin carpet cloak, we set $\Gamma \rightarrow 1$ which leads to $\alpha \rightarrow 0$. The diagonal form of Eq. (1) is obtained by rotating the matrix around its principal axis i.e. z-axis at the angle of $\varphi =tan^{-1}(f'(x))+\pi /2$^17^. Therefore, the diagonal form of Eq. (1) is expressed as

2 $$\begin{aligned} \frac{\overline{\overline{\varepsilon '_d}}}{\varepsilon }=\frac{\overline{\overline{\mu '_d}}}{\mu }= \begin{bmatrix} \ \alpha &{} 0&{} 0 \\ 0 &{} \frac{1+(f'(x))^2}{\alpha } &{} 0\\ 0 &{} 0 &{} \frac{1}{\alpha } \end{bmatrix} \overset{\alpha \rightarrow 0}{\mathop =}\, \begin{bmatrix} \ 0 &{} 0&{} 0 \\ 0 &{} \infty &{} 0\\ 0 &{} 0 &{} \infty \end{bmatrix} \end{aligned}$$

The material achieved by Eq.  (2) is called an IAM with the optical axis along the $x'$ direction. Therefore, by considering Eq. (2) and the rotation angle $\varphi$, one can conclude that an ideal ultrathin carpet cloak is composed of the IAM whose optical axis is perpendicular to the object’s surface, which is demonstrate in Fig. 1 by $\hat{n}$.

### Numerical simulation

**Figure 2:**
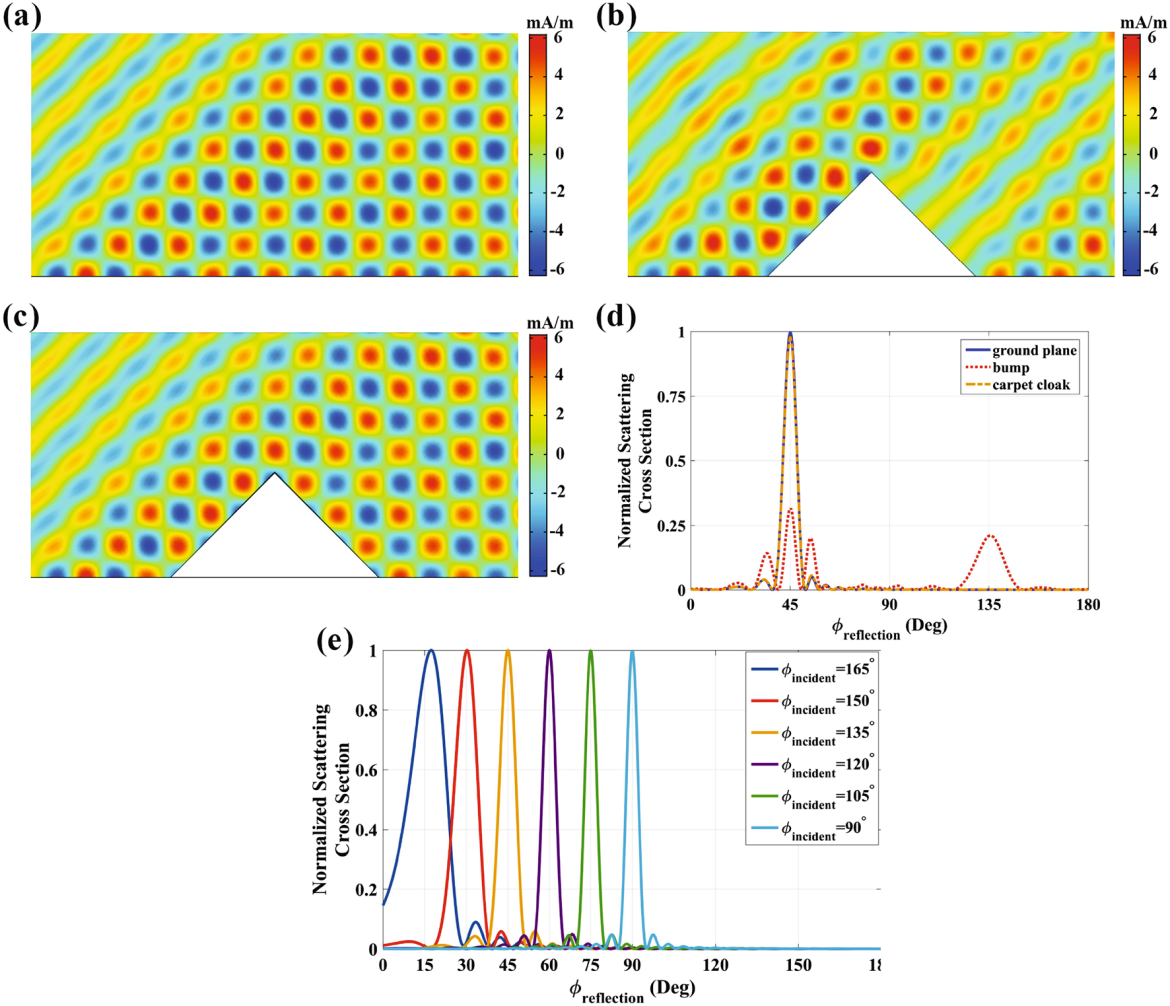
Performance of the invisibility carpet cloak based on IAM for a triangle shape object with $\alpha =0.01$. Total magnetic field distribution at $\phi _{inc}=135^\circ$ for (**a**) flat ground plane, (**b**) triangle bump and (**c**) invisibility carpet cloak. (**d**) Far-field pattern of corresponding cases. (**e**) Far-field patterns of carpet cloak at $\phi _{inc}=90^\circ$, $\phi _{inc}=105^\circ$ ,$\phi _{inc}=120^\circ$, $\phi _{inc}=135^\circ$,$\phi _{inc}=150^\circ$, and $\phi _{inc}=165^\circ$.

In the following, we carry out full-wave simulations using COMSOL Multiphysics finite element solver to demonstrate the functionality of ultrathin carpet cloaks to conceal arbitrary shape objects from all viewing angles. We assume the structure is illuminated by a TM-polarized (*H* along the *z* direction) plane wave at *f*=3.5 GHz. The first example is dedicated to a triangle object with the base and height parameters of 0.2 m and 0.4 m, respectively. Since, selecting the exact value of 0 for $\alpha$ causes some errors in the simulation process, we set $\alpha =0.01$. Figure 2a–c illustrate the near field distributions of the total magnetic field for a ground plane, an uncovered bump and the object covered with the proposed IAM. As can be seen, by comparing Fig. 2a,c, the carpet cloak has a perfect mirror-reflection behavior. However, the triangle bump without a cloaking shell scatters the incident wave into free space in directions other than specular reflection. Moreover, the far-field pattern of these three cases are compared in Fig. 2d under the incident wave angle of $\phi _{inc}=135^\circ$. As demonstrated, the far-field pattern of an alone bump has a significant backward scattering, while the cloaking structure is exactly similar to the ground plane, which indicates its ideal performance. To further demonstrate the effectiveness of the proposed ultrathin cloak, the structure is examined under other arbitrary incident wave angles $\phi _{inc}=90^\circ$, $\phi _{inc}=105^\circ$ ,$\phi _{inc}=120^\circ$,$\phi _{inc}=150^\circ$, and $\phi _{inc}=165^\circ$. The scattering cross section of these mentioned cases are plotted in Fig. 2e, where a single beam is noticed at the desired angle for each case. The difference of their beam width comes from the fact that the more oblique the incident wave, the smaller the equivalent aperture size^18^. Consequently,the presented IAM can hide objects from all range of incident angles while having an extremely thin profile in comparison to the object size.

**Figure 3:**
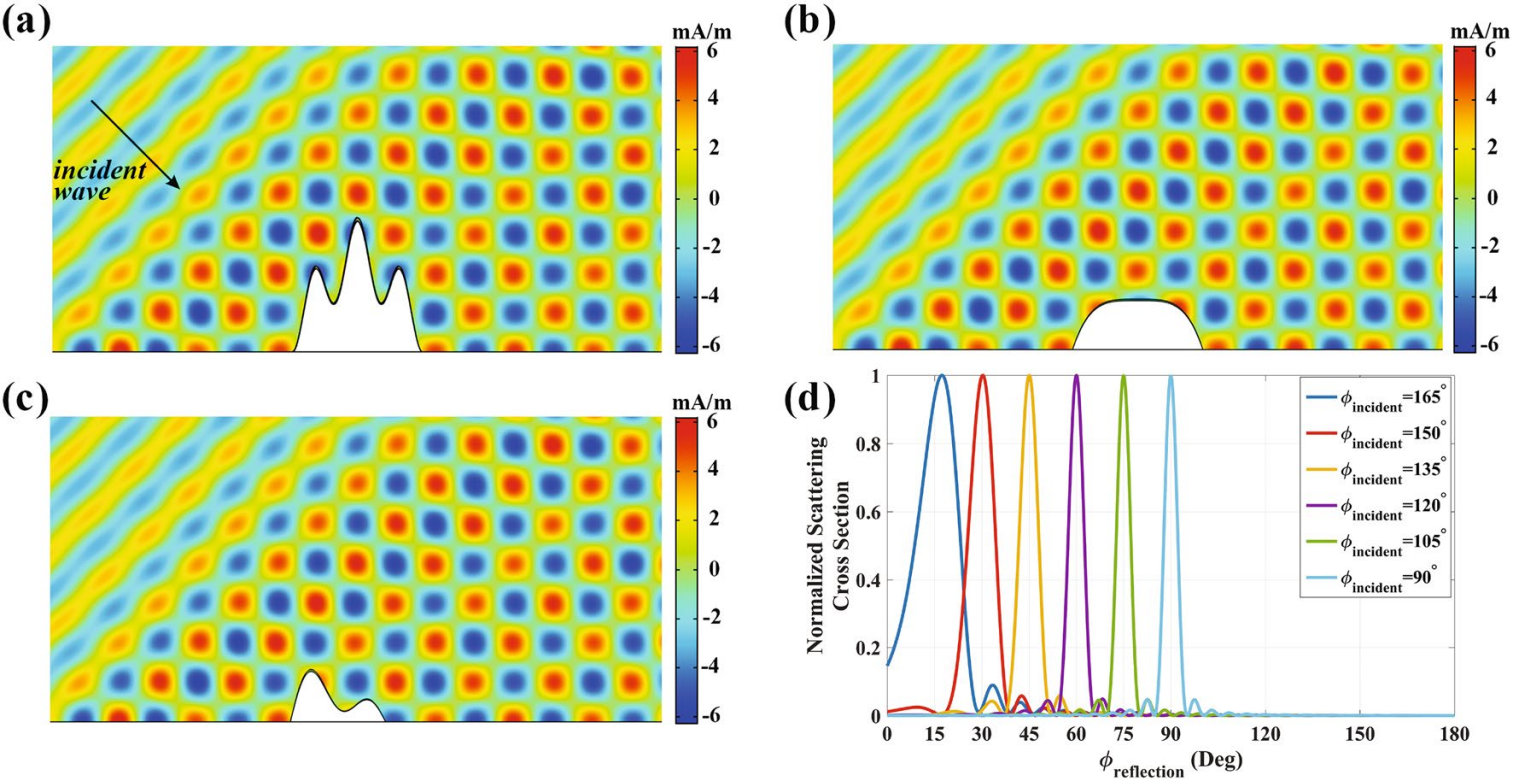
Performance of the invisibility carpet cloak based on IAM for an arbitrary shape object with $\alpha =0.03$. (**a**–**c**) Total Magnetic field distribution at $\phi _{inc}=135^\circ$ . (**d**) Far-field patterns at $\phi _{inc}=90^\circ$, $\phi _{inc}=105^\circ$ ,$\phi _{inc}=120^\circ$, $\phi _{inc}=135^\circ$,$\phi _{inc}=150^\circ$, and $\phi _{inc}=165^\circ$.

Moreover, for the objects with arbitrary shape that are important in the practical scenarios, the previously reported approaches are not applicable due to the necessity to inhomogeneous and shape-dependent materials. Nevertheless, the material introduced in Eq. (2), can mitigate most of these drawbacks. To this aim, an arbitrary shape object is coated by a thin IAM layer with $\alpha =0.03$. As illustrated in Fig. 3a, the total magnetic field distribution of the concealed object is identical to that of the ground plane as if the object does not exist. For a better comparison, please refer to Fig. 1a. In order to ensure comprehensive coverage, we have incorporated additional simulations. These simulations aim to further illustrate the independence of the proposed method from the cloak’s geometry. We employed the same materials to fabricate various cloaks with arbitrary cross-sections. The results of these simulations are presented in Fig. 3b,c. As depicted in the aforementioned results, the functionality of the obtained materials remains consistent regardless of the input geometry. It is demonstrated that the total magnetic field of the concealed arbitrary objects exhibits complete similarity to the scenario in free space. Moreover, in contrast to the previous metasurface cloaks, when the angle of incidence varies, the functionality of our designed cloak remains unchanged, as validated with the scattering cross section in Fig. 3d. In other words, the functionality of the cloak presented here is not restricted to a specific range of incident wave angles.

Although the propounded cloak can conceal the objects with ultrathin coating layer from all viewing angles, this challenging question is raised that how an IAM could be realized in practice. To answer this question, we will take the advantage of EMT so as to validate the performance of an implemented triangle shape carpet cloak. With the assumption of TM polarization, the only important parameters are $\varepsilon _{xx}=\alpha$, $\varepsilon _{yy}={1+(f'(x))^2}/\alpha$ and $\mu _{zz}=1/\alpha$. As discussed above, when $\alpha \rightarrow 0$, the IAM with $\varepsilon =diag[0,\infty ]$ and $\mu =\infty$ is acquired where $diag[\cdot ]$ represents a diagonal matrix.

## Implementation

Although the propounded cloak can conceal the objects with ultrathin coating layer from all viewing angles, this challenging question is raised that how an IAM could be realized in practice. To answer this question, we will take the advantage of EMT so as to validate the performance of an implemented triangle shape carpet cloak. With the assumption of TM polarization, the only important parameters are $\varepsilon _{xx}=\alpha$, $\varepsilon _{yy}={1+(f'(x))^2}/\alpha$ and $\mu _{zz}=1/\alpha$. As discussed above, when $\alpha \rightarrow 0$, the IAM with $\varepsilon =diag[0,\infty ]$ and $\mu =\infty$ is acquired where $diag[\cdot ]$ represents a diagonal matrix.

**Figure 4:**
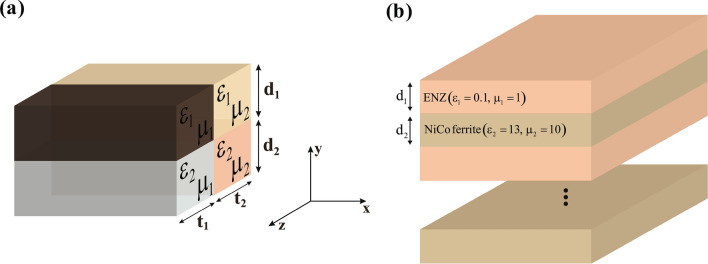
Proposed multi-layered structures that mimic the IAM. (**a**) Four layer structure, as the electric permittivity and magnetic permeability are changed periodically in *y* and *z* directions, respectively. (**b**) Two layer structure, with the periodicity in *y* direction.

Theoretically, according to EMT, the multi-layered structure displayed in Fig. 4a that consists of four isotropic blocks with constitutive parameters $\varepsilon _{1,2}=\pm 1$, $\mu _{1,2}=\pm 1$, and $d_1=d_2$, $t_1=t_2$ can behave as an anisotropic medium with the parameters of IAM, if the thickness of each layer is much smaller than the operative wavelength. To construct each of these layers, a composite of resonance metamaterials should be utilized. Due to the coupling effect between adjacent blocks, the implementation procedure faces challenge in practice. To meet this challenge, we use high permeability material as discussed in the following.

In general, for the layered structure shown in Fig. 4b we have the following effective parameters:

3 $$\begin{aligned}&\varepsilon _{xx}=f\varepsilon _1+(1-f)\varepsilon _2 \\ \nonumber&\frac{1}{\varepsilon _{yy}}=\frac{f}{\varepsilon _1}+\frac{1-f}{\varepsilon _2} \\ \nonumber&\mu _{zz}=f{\mu _1}+(1-f){\mu _2} \end{aligned}$$

in which $$f=d_1/(d_1+d_2)$$.

**Figure 5:**
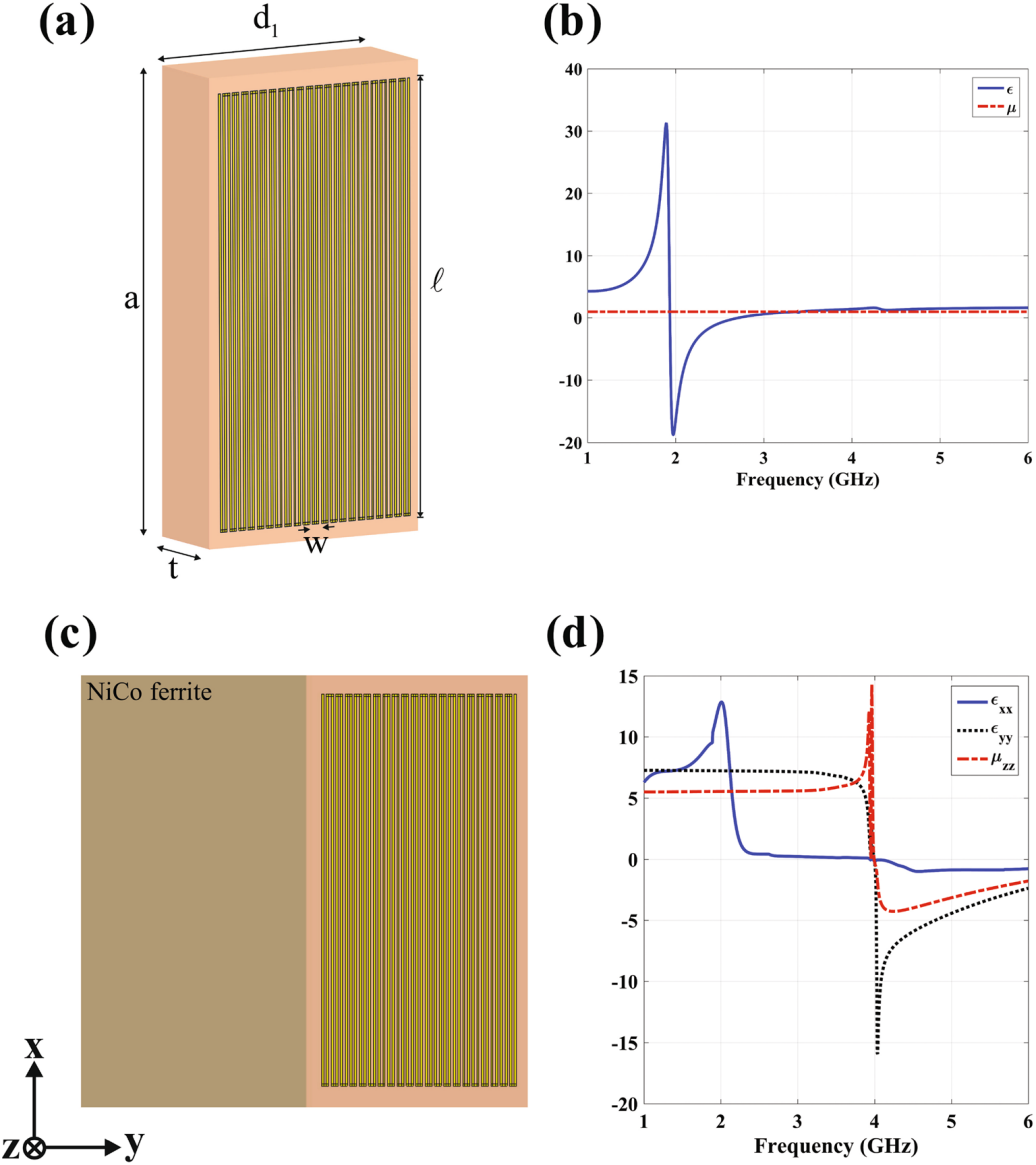
(**a**) Composite of SR-meander line. (**b**) The retrieved materials for $\varepsilon =0.1$, $\mu =1$ with the desired characteristics at f=2.7 GHz. (**c**) The final composition of IAM made of ENZ and NiCo ferrite with parameters of $t=1.2$ mm, $d_1=3$ mm, $a=6$ mm, $l=5.6$ mm, $w=0.1$ mm. (**d**) The retrieved materials which the desired characteristics occurred at f=3.5 GHz.

As proof of principle, a triangle shape carpet cloak with $\alpha =0.18$ is implemented through the EMT. The required constitutive materials are obtained as $\varepsilon _{xx}=0.15$, $\varepsilon _{yy}=6.46$ and $\mu _{zz}=5.56$ after the diagonalization. Based on Eq. (3), one can implement this coating layer by using an alternate structure introduced in Fig. 5a which consists of NiCo ferrite ($\varepsilon _{r}=13$, $\mu _{r}=10$)^19^ and ENZ ($\varepsilon _{r}\simeq 0.1$, $\mu _{r}=1$). To achieve the ENZ, the geometry of meander line unit cell on the RO4003 substrate is optimized (see Fig. 5a)^20,21^. The ENZ is designed and simulated with CST Microwave Studio commercial software, where its retrieved material parameters is plotted in Fig. 5b. The periodic boundary conditions are applied to the x and z directions, and the Floquet ports are assigned to the y-direction. Two metamaterial layers are used in the direction of propagation to consider the effect of coupling between adjacent unit cells^22,23^. The calculated S-parameters are then employed to retrieve the material propertie with the anisotropic parameter retrieval algorithm^24^. As a result, the behavior of the metamaterial has been obtained at all incident angles. However, when the ENZ is placed in vicinity of NiCo ferrite layer (see Fig. 5c), its electromagnetic characteristics are changed a little. After sweeping the geometrical parameters of ENZ layer, the final realized IAM with the retrieved parameters of $\varepsilon _{xx}=0.15$, $\varepsilon _{yy}=6.96$ and $\mu _{zz}=5.71$ is achieved as presented in Fig. 5d. As can be seen, from the retrieved parameters, the designed metamaterials are capable of possessing the appropriate values at the desired frequency 3.5 GHz. It is important to note that, similar to the TM-polarization scenario, where the combination of ENZ and high-epsilon-mu layers is employed, for TE-polarizations, we should utilize mu-near-zero (MNZ) and high-epsilon-mu layers. This conclusion is derived from the mathematical result of TE version of Equation (3). To provide an illustrative example, in order to implement the same triangle cloak, we can employ a NiCo ferrite layer as the high-epsilon-mu material, along with a metamaterial layer characterized by the parameters $\varepsilon _{r}=-7.6$, $\mu _{r}=0.05$, serving as the MNZ layer.

**Figure 6:**
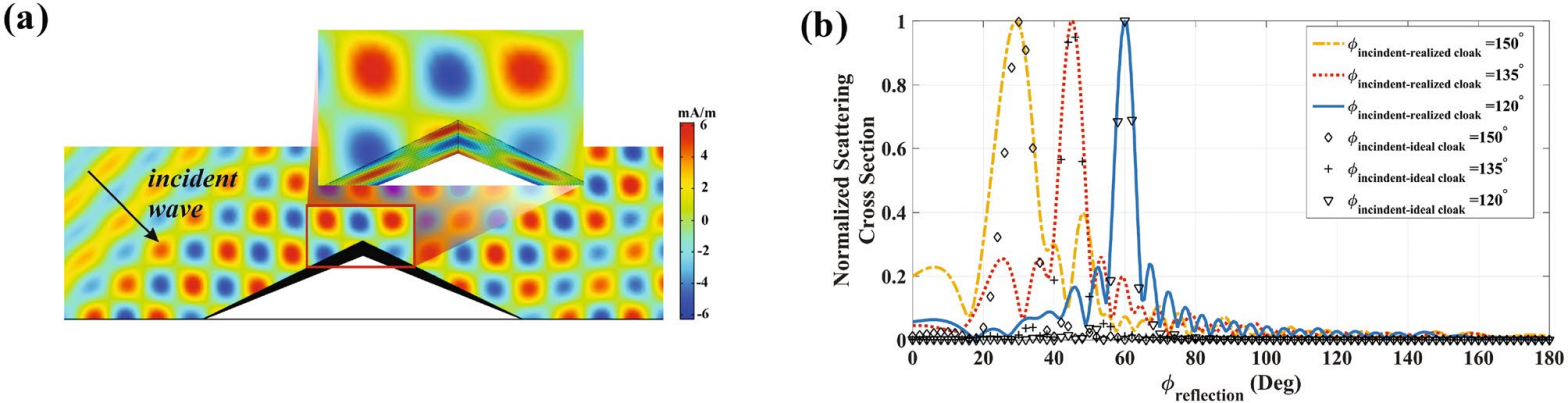
Performance of the realized carpet cloak for a triangle shape object with $\alpha =0.18$. (**a**) Magnetic field distribution at $\phi _{inc}=135^\circ$ . (**b**) Far-field patterns for the two case of ideal cloak and realized at $\phi _{inc}=120^\circ$, $\phi _{inc}=135^\circ$ and $\phi _{inc}=150^\circ$.

The triangle shape carpet cloak with $\alpha =0.18$ and ${f}'=0.4$ is realized with the retrieved parameters of the propounded unit cell (see Fig. 6a). The metamaterial blocks are parallel to the object surface which is shown in the figure inset. As illustrated in Fig. 6b, the designed metamaterials are capable of mimicking the IAM behavior and concealing the object from all viewing angles, affirming the successful realization. The occurrence of a sub-flap in Fig. 6b can be attributed to the approximations made during the implementation process. As noted before, the determined parameters of the proposed metamaterial exhibit a discrepancy of 10% approximately compared to the desired constitutive materials. While this variation can generally be disregarded for most incident angles, it becomes significant at extremely oblique angles, leading to the generation of undesired scattering beams. Nonetheless, it should be noted that the side-lobe level of the scattering cross section is approximately 8 dB, and it is possible to enhance this by modifying the geometry of the metamaterial building block.

**Figure 7:**
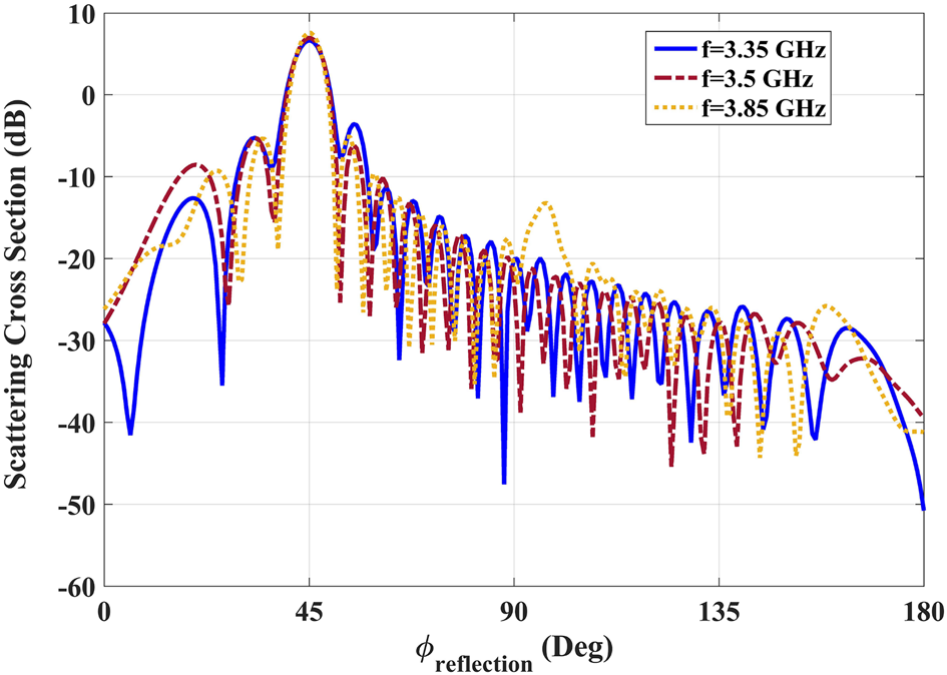
Scattering cross section of the realized cloaking structure in the frequency of 3.35 GHz, 3.5 GHz and 3.85 GHz..

Furthermore, the performance of the structure was assessed within the frequency range of 3.35 GHz to 3.85 GHz, and the corresponding results are presented in Fig. 7 for three specific frequencies. It is evident that throughout the entire frequency band, the main reflected beam remains at $\phi =45^\circ$, while the sidelobe level exceeds 10 dB. Consequently, the cloaking performance exhibits a bandwidth of 0.5 GHz. This observation aligns with the fact that the retrieved parameters of the implemented infinitely anisotropic medium (IAM) exhibit a flat response around the desired frequency. As a result, a broadband stealth performance is successfully achieved. It is noteworthy to acknowledge that in the context of practical applications, the rotation of the presented 2D cloak along the y-axis yields a 3D cylindrical ground-plane cloak as indicated in ^25^. By virtue of the rotational symmetry exhibited by the 3D cloak, it effectively conceals the concealed area from all viewing angles, including azimuth and elevation perspectives. In order to enhance the capabilities of the cloak in the optical frequency range, a potential strategy involves the utilization of a multi-layered structure. This structure incorporates negative metamaterials as illustrated in Fig. 4. To achieve this, it is necessary to employ metamaterials that are specifically engineered for optical frequencies. Extensive research has been conducted on optical metamaterials, and their characteristics and potential applications have been extensively explored in various scholarly articles^26,27^.

## Discussion

In conclusion, in this paper, we have presented a new material based on coordinate transformation capable of obviating the conventional challenges of carpet cloaks. The attained material which is called IAM, makes the carpet cloak with arbitrary shape extremely thin while the cloaking functionality remain unchanged for all viewing angles. Numerical simulations are proposed corroborating well the validity and effectiveness of the propounded approach. In addition, the metamaterial building blocks that are competent to mimic the behavior of an IAM are proposed and designed. Then, as a proof of concept, a triangle shape carpet cloak is realized with the aid of EMT and the designed metamaterials. It is observed that the simulation results exhibit good agreement with the theoretical predictions, which corroborates the correctness of the designed procedure. We believe that the newly proposed material in this paper constitutes a significant step towards the invisibility cloaks.

## Data Availability

The datasets used and/or analyzed during the current study available from the corresponding author on reasonable request.
